# Synthesis, density functional theory study and in vitro antimicrobial evaluation of new benzimidazole Mannich bases

**DOI:** 10.1186/s13065-020-00697-z

**Published:** 2020-07-25

**Authors:** Maria Marinescu, Ludmila Otilia Cinteză, George Iuliu Marton, Mariana-Carmen Chifiriuc, Marcela Popa, Ioana Stănculescu, Christina-Marie Zălaru, Cristina-Elena Stavarache

**Affiliations:** 1 Department of Organic Chemistry, Biochemistry and Catalysis, Faculty of Chemistry, University of Bucharest, Bucharest, 050663 Romania; 2 Department of Physical Chemistry, Faculty of Chemistry, University of Bucharest, Bucharest, 030018 Romania; 3 Faculty of Applied Chemistry and Materials Science, University “Politehnica” of Bucharest, 1-7 Polizu, 011061 Bucharest, Romania; 4 Department of Botanic-Microbiology, Faculty of Biology, University of Bucharest, 1-3 Aleea Portocalilor, 60101 Bucharest, Romania; 5 Research Institute of the University of Bucharest, 91-95 Splaiul Independentei, 050095 Bucharest, Romania; 6 Institute of Organic Chemistry “C.D. Nenitzescu” of the Romanian Academy, 202B Splaiul Independentei, 060023 Bucharest, Romania

**Keywords:** Benzimidazole, Mannich base, Synthesis, DFT study, Antimicrobial activity, Biofilm

## Abstract

The tri-component synthesis of novel chiral benzimidazole Mannich bases, by reaction between benzimidazole, aqueous 30% formaldehyde and an amine, the biological evaluation and DFT studies of the new compounds are reported here. The ^1^H-NMR, ^13^C-NMR, FTIR spectra and elemental analysis confirm the structures of the new compounds. All synthesized compounds were screened by qualitative and quantitative methods for their in vitro antibacterial activity against 4 bacterial strains. DFT studies were accomplished using GAMESS 2012 software and HOMO–LUMO analysis allowed the calculation of electronic and structural parameters of the chiral Mannich bases. The geometry of 1-methylpiperazine, the cumulated Mullikan atomic charges of the two heteroatoms and of the methyl, and the value of the global electrophilicity index (ω = 0.0527) of the **M-1** molecule is correlated with its good antimicrobial activity. It was found that the presence of saturated heterocycles from the amine molecule, 1-methyl piperazine and morpholine, respectively, contributes to an increased biological activity, compared to aromatic amino analogs, diphenylamino-, 4-nitroamino- and 4-aminobenzoic acid. The planarity of the molecules, specific bond lengths and localization of HOMO–LUMO orbitals is responsible for the best biological activities of the compounds.

## Introduction

Mannich bases result from a three-component condensation between a substrate with acidic hydrogen, an aldehyde and an amine. Heterocycles Mannich bases are remarkable compounds with various medicinal properties such as: antimicrobial [1–8], anticancer [9, 15], antiviral [9, 10], analgesic [11–13], anticonvulsant [2, 10] anti-inflammatory [11–14], anti-HIV [2, 10], antimalarial [15], anti-Alzheimer [16, 17], anthelmintic [15], antioxidant [16, 17] and so forth. Recent studies show Mannich bases as multifunctional agents against Parkinson disease, with good in vitro anti-inflammatory and neuroprotective effects [18]. Also, 1,2,4-triazole-adamantyl N-Mannich bases were reported for their in vivo hypoglycemic and in vitro antimicrobial activities [19].

Benzimidazole scaffold is a key pharmacophore in modern drug discovery [20], and its derivatives represent important bioactive molecules [6] with privileged structures in medicinal chemistry [21]. This can be very well confirmed by the increasing number of synthesized compounds which contain the benzimidazole moiety, with a wider range of therapeutic properties [21, 22] as well as by the attempt to synthesize benzimidazole compounds with preferential geometries possessing certain biological properties [22, 23].

**Fig. 1:**
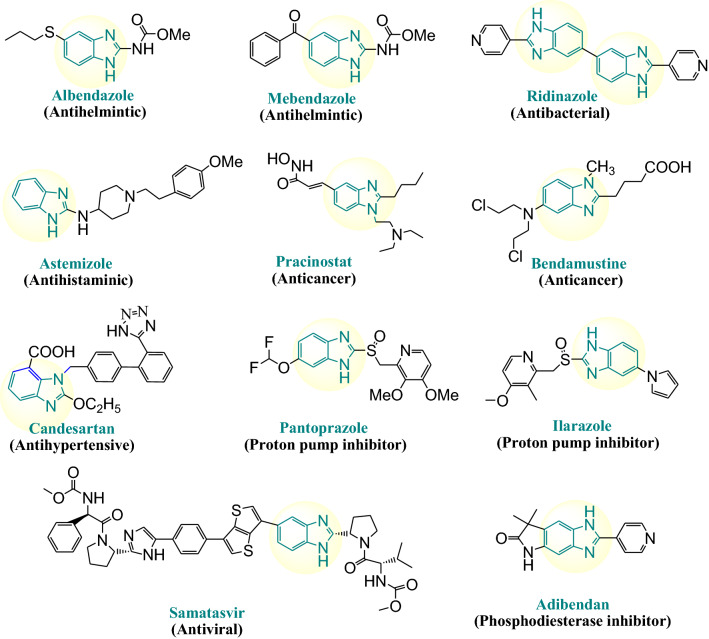
The structures of some representative benzimidazole based drugs

A large number of benzimidazole compounds have been employed as candidates for the treatment of various types of diseases or as clinical drugs, including anticancer agents (Pracinostat, Bendamustine), antihistamine agents (Astemizole), anthelmintic agents (Albendazole, Mebendazole) [20, 22], antibacterial agents (Ridinazole), antihypertensive agents (Candesartan), proton pump inhibitors (Pantoprazole, Ilaprazole), antiviral agents (Samatasvir) and phosphodiesterase inhibitors (Adibendan) (Fig. 1).

Benzimidazole derivatives developed a considerable interest in medical domain due to their therapeutic action as antitumor [24–33], antimicrobial [6, 34–38], anthelmintic [39, 40], proton pump inhibitors [22, 41], anti-inflammatory [42, 43] and anti-hypertensive [44] drugs. Astemizole-related compounds demonstrated anti-prion activity for treatment of Creutzfeldt-Jakob disease, while albendazole compounds are currently used as medication for the treatment of a variety of parasitic worm infestations. Benzimidazoles treat mitochondrial dysfunction in Alzheimer disease [45], possess neurotropic, psychoactive, analgesic effects [46], anticoagulant proprieties [47] and are efficient agents in diabetes mellitus [48]. Benzimidazole derivatives were reported as potential EGFR and erbB2 inhibitors [49, 50], DNA/RNA binding ligands [51, 52] and antiquorum-sensing agents [53].

Chiral benzimidazole derivatives with excellent preclinical in vitro ADME were screened as Na_V_1.8 (Voltage-gated sodium channels) blockers [54]. Benzimidazole compounds proved to be anti-HIV-1 agents through the protection of APOBEC3G protein [55]. Antioxidant activity was reported for benzimidazoles grafted with aromatic nuclei [56].

Recent literature mentions a series of syntheses of Mannich chiral bases, predominantly by classical procedures and less by green methods. Referring to classical procedures, Bernardi et al. studied the catalytic asymmetric Mannich reactions for the synthesis of optically active α,β-diamino acid derivatives [57] while Ibrahem and Guo groups used the chiral aminoacids for the direct asymmetric three-component Mannich reaction [58, 59]. Bhadury and Song reviewed the mechanism and the stereochemistry of organocatalytic asymmetric Mannich reactions [60], Cai and Xie provide an overview of asymmetric Mannich reactions with different organocatalysts, such as: chiral Brønsted acids, chiral amines, chiral bifunctional thiourea, and others [61]. In recent years, the stereoselective asymmetric Mannich reactions of aldehydes catalyzed by chiral primary amine were reported by Dai et al. [62], enantioselective Mannich syntheses promoted by chiral phosphinoyl-aziridines [63], asymmetric Mannich reactions to generate chiral β-amino esters [64], and copper-catalyzed enantioselective Mannich reactions of N-acylpyrazoles and isatin [65].

Among the green methods of asymmetric Mannich syntheses, we can mention protease-catalysed direct reaction in acetonitrile [66], N,N’-dioxide metal complexes-catalysed reaction in dichloromethane [67] and synthesis of β-amino carbonyl compounds using maleic acid in ethanol [68].

Antibiotics have been considered one of the most important discoveries in the early part of the last century, being very effective in controlling bacterial infections, but their inappropriate use has rapidly led to the emergence of antibiotic-resistant pathogens. Resistance to antibiotics has been increasing in recent years and becoming a serious and global challenge to the drug discovery [69]. Nowadays there are bacteria already resistant to nearly all available antibiotics. Therefore, there is a growing interest in the finding of new, effective antibiotics [70, 71].

It was stated that the planarity of the compound and symmetry of the molecule are advantages for a high antimicrobial activity [6]. Other authors find that low polarity of molecules is an advantage for antimicrobial activity [37]. Also, antimicrobial activity is correlated with the presence of functional groups such as, benzimidazole esters as antifungal agents [72] and amino for antimicrobial activity [73].

Based on our previous findings on good microbial activity, correlated with compound structure [6], in this study propose directed synthesis of some benzimidazole Mannich bases, the screening of their antimicrobial activity, density functional theory study (DFT) on the synthesized structures and discussions on drug-design (Additional file 1).

## Results and discussion

**Fig. 2:**
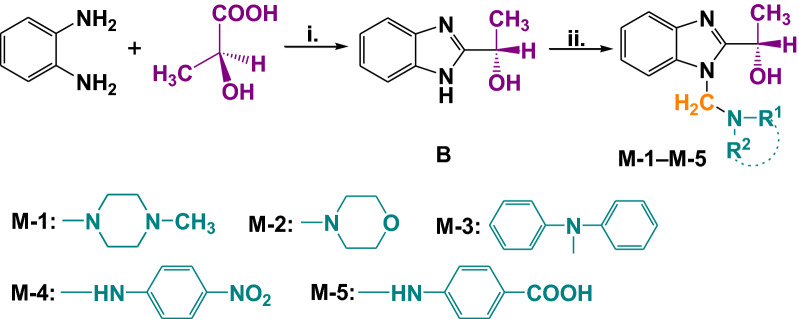
Synthetic pathways for Mannich bases **1**–**5**; Reagents and conditions: i. HCl 4N, 2 h at 140°, NH_3_; ii. CH_2_O, HNR^1^R^2^, HCl, EtOH, 3 h reflux under stiring

**Fig. 3:**
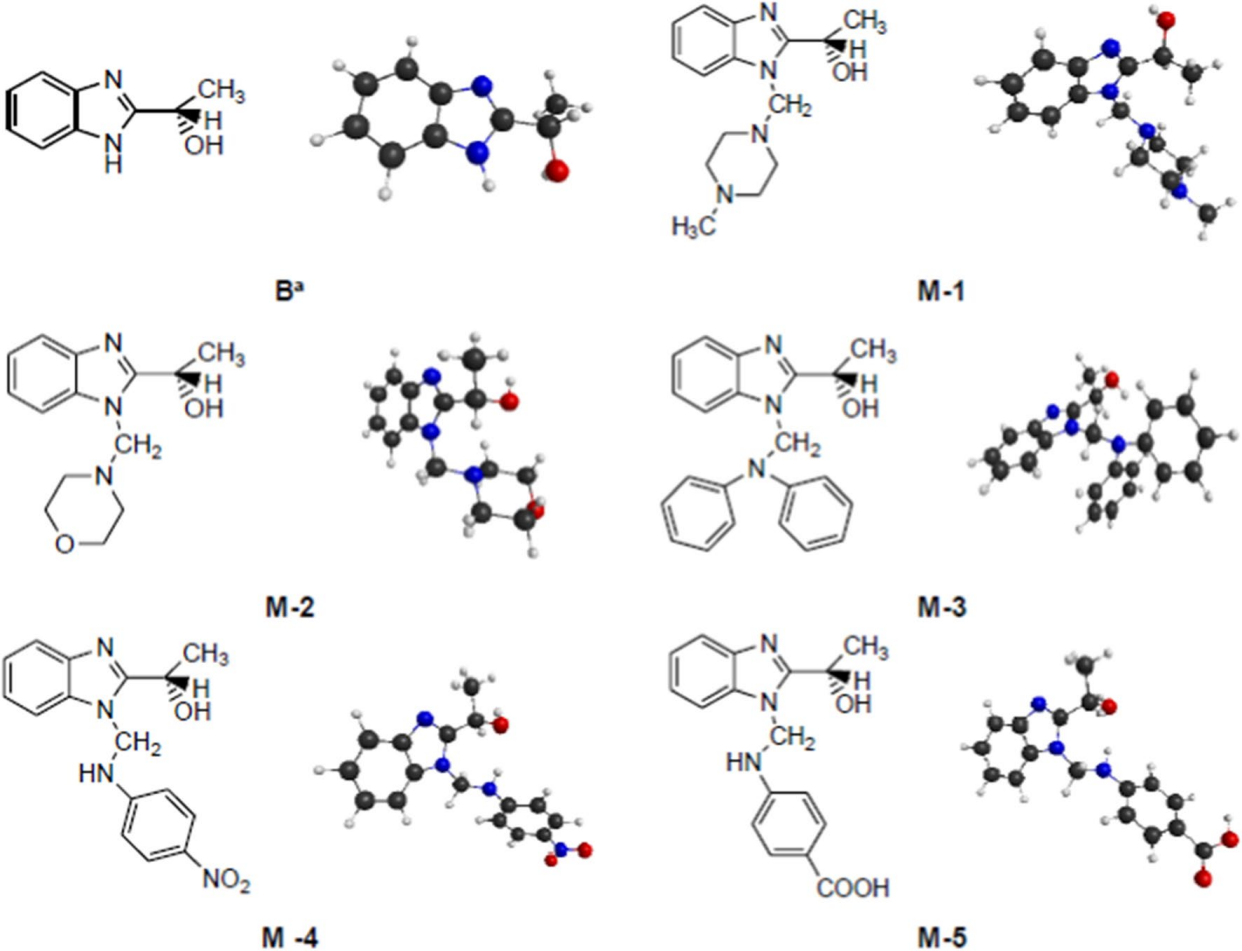
The structure formulas and the DFT optimized structures of benzimidazole **B** and Mannich bases **M-1**–**M-5** (black-C; grey-H; red-O; blue-N). For interpretation of the references to colour in this figure legend, the reader is referred to the web version of this article. ^a^From Ref. [6]

The synthesis of the new (*S*)-1-(1(alkylaminomethyl)-benzo[d]imidazole-2-yl)ethanol is shown in Fig. 2. (*S*)-1-(1*H*-Benzo[d]imidazole-2-yl)ethanol **B** was synthesized according to the procedure described in our previous work [6]. The novel bases **M**-**1–M-5** (Fig. 3) were obtained by the classical Mannich reaction, at reflux temperature, as a three component condensation between benzimidazole **B**, aqueous 30% formaldehyde and an amine reagent [7]. The syntheses afford good yields (73–83%). The hydrogen atom of the imino group of benzimidazole **B** is quite active to participate in Mannich reactions.

In the Mannich reaction, primary or secondary amines are nucleophilic reactants for the carbonyl group of the formaldehyde [7] and the Mannich bases **M-1–M-5** or β-amino-carbonyl compounds are the final products of this condensation reaction.

Determination of the optical purity of compounds **B**, **M-1**–**M-5** was done by HPLC analysis using a CHIRALPAK column. All the benzimidazoles were found to have enantiomeric excess > 90%. All benzimidazole structures were confirmed by spectroscopic methods: ^1^H-NMR, ^13^C-NMR, FTIR, MS and elemental analysis. The main features of the ^1^H-NMR spectra of the compounds **M-1–M-5** are the signals corresponding to the methylene protons directly bonded to both amine nitrogen atom and heterocyclic nitrogen atom which appears in the range 5.1–6.01 ppm [74, 75]. The most shielded methylene protons were observed in case of **M-3** Mannich base due to the simultaneous presence of the two aromatic nuclei, linked by the amine group. The hydrogen atoms in the benzimidazole moiety appear at the expected values of 7.11–7.74 ppm. The presence of substituents at the nitrogen atom of the Mannich bases does not greatly affect the chemical shifts of benzimidazole aromatic protons. The proton of the hydroxyl group constantly appears at 5.72–5.79 ppm and the methine group—as a quartet—at 4.92–4.94 ppm.

The ^13^C-NMR spectra confirm the structure of the Mannich bases obtained by amino alkylation. The methyl group of 2-hydroxyethylbenzimidazole appears at 21–23 ppm. The most shielded aliphatic signal corresponding to Mannich methylene group appears at 61 ppm for the Mannich bases obtained with aliphatic amines **M-1** and **M-2** and at around 81 ppm for the Mannich bases with aromatic amines **M-3**–**M-5**. The aromatic carbon atoms in the benzimidazole moiety appear in the expected ranges.

The MS spectra of all Mannich bases confirmed the presence of the molecular ions.

FTIR spectra show the stretching bands characteristic to the tertiary aromatic amines in the range 1143–1360 cm^−1^ for the compounds **M-1**–**M-5**. The presence of a methylene Mannich group was confirmed by a strong signal in range of 1457–1530 cm^−1^ of the bending vibration δ(CH_2_). A broad band in the range 3200–3570 cm^−1^ was assigned to ν(OH) stretching vibration. The stretching bands due of the benzimidazole ring can be found at 742–753 cm^−1^, 1108–1213 cm^−1^ and 2829–3085 cm^−1^ range.

### Antibacterial activity

The antibacterial activity of the benzimidazole compounds has been evaluated against three bacterial strains, both Gram positive cocci, *Staphylococcus aureus ATCC 6538* (*S. aureus*) and Gram negative bacilli: *Pseudomonas aeruginosa ATCC 27853* (*P. aeruginosa*) and *Escherichia coli ATCC 8739* (*E. coli*) [6, 72] by comparing their minimum inhibitory concentration (MIC) values against a standard drug, erythromycin.

In the qualitative assay (Table 1), from the tested compounds, the most active proved to be **M-1**, active against all four tested strains, followed by the Mannich bases **M-2**, **M-3** and benzimidazole **B**, active against three of the 4 tested strains, all inhibited the growth of Gram negative strains and fungus. All compounds except **M-5** have also antifungal effects. Compounds **M-4** and **M-5** possess a much lower antimicrobial activity, the first one only antifungal activity and the second one antibacterial activity against one Gram negative bacilli strain, *P. aeruginosa*.

**Table 1:** The antimicrobial activity of the tested compounds expressed semi-quantitatively, as the absence of the microbial growth (−), slight decrease of the microbial growth (±), total inhibition of microbial growth (+, ++)

Compound/Microbial tested strain	B	M-1	M-2	M-3	M-4	M-5	Erythromycin	Clotrimazole
*S. aureus* ATCC 6538	±	+	±	±	–	±	+	n/a
*P. aeruginosa* ATCC 27853	+	+	++	+	–	+	++	n/a
*E. coli* ATCC 8739	+	+	+	++	±	±	++	n/a
*C. albicans* ATCC 10231	+	+	+	++	+	–	n/a	++

All the MIC values obtained for the compounds (**B**, **M-1**-**M-5**) are reported in Table 2. It was observed that the DMSO solvent does not influence the antibacterial activity of the tested compounds at the working concentrations of 0.25, 0.062, 0.031, 0.01562, 0.008 and 0.002 μg mL^−1^.

**Table 2:** The MIC (μg mL^−1^) values of the tested compounds against the tested microbial strains

Compounds	*S. aureus* ATCC 6538	*P. aeruginosa* ATCC 27853	*E. coli* ATCC 8739	*C. albicans* ATCC 10231
**B**	< 1	0.008	< 1	0.002
**M-1**	< 1	0.031	0.031	0.031
**M-2**	0.052	< 1	< 1	0.032
**M-3**	< 1	< 1	< 1	0.052
**M-4**	0.030	< 1	<1	0.030
**M-5**	< 1	< 1	< 1	< 1
Erythromycin	0.062	0.062	0.062	n/a
Clotrimazole	n/a	n/a	n/a	0.031

From the tested compounds, the most active proved to be **M-1**, active against two of three tested strains, followed by the Mannich bases **M-2**, **M-4** and benzimidazole **B**, active against one of the 3 tested strains. Compound **B** exhibited the most significant activity against *Pseudomonas aeruginosa* (MIC 0.008 μg mL^−1^) and **M-1** was found to be twice as active as the standard (MIC 0.031 μg mL^−1^). Compound **M-4** was two fold active compared to the standard (MIC 0.030 μg mL^−1^) and compound **M-2** was slightly better than the standard (MIC 0.052 μg mL^−1^) against *Staphylococcus aureus* (MIC 0.062 μg mL^−1^). Compound **M-1** was also much better than the standard (MIC 0.031 μg mL^−1^) against *Escherichia coli*.

Experimental, a similar behaviour as compared to their microbicidal properties was revealed by the investigation of the anti-biofilm activity of the benzimidazole compounds. (Table 3). Compounds **B** and **M-1** were two fold active compared to the standard against *Pseudomonas aeruginosa* (MBEC 0.01562 μg mL^−1^) while **M-5** was as active as the standard. Compound **M-4** was found to be more active compared to the standard (MBEC 0.031 μg mL^−1^) while **M-2** was eight times less active than the standard (MBEC 0.25 μg mL^−1^) against *Staphylococcus aureus*. Only compound **M-1** was active against *Escherichia coli* but eight times less active than the standard (MBEC 0.25 μg mL^−1^).

**Table 3:** The MBEC (μg mL^−1^) values of the tested compounds against the tested microbial strains

Compounds	*S. aureus* ATCC 6538	*P. aeruginosa* ATCC 27853	*E. coli* ATCC 8739	*C. albicans* ATCC 10231
**B**	< 1	0.01562	< 1	0.034
**M-1**	< 1	0.01562	0.25	0.25
**M-2**	0.25	< 1	< 1	0.032
**M-3**	< 1	< 1	< 1	< 1
**M-4**	0.031	< 1	< 1	0.030
**M-5**	< 1	0.031	<1	< 1
Erythromycin	0.062	0.062	0.062	n/a
Clotrimazole	n/a	n/a	n/a	0.031

### Antifungal activity

The synthesized compounds **B**, **M-1**-**M-5** were also screened for in vitro antifungal activity against *Candida albicans* ATCC 10231 fungal strain. Here, Clotrimazole was used as the standard drug. All the noticed MIC values are listed in the Table 2. Compound **B** was 15 times more active than the standard (MIC 0.002 μg mL^−1^), while **M-1**, **M-2** and **M-4** were almost as active as the standard (MIC 0.031 μg mL^−1^) and **M-3** was almost half active against the standard (MIC 0.052 μg  mL^−1^). A slightly different behavior was determined for anti-biofilm activity. Compounds **B**, **M-2** and **M-4** were almost as active as the standard (MIC 0.031 μg mL^−1^) and compound **M-1** was almost eight times less active than standard.

A better antifungal activity than the antimicrobial activity is indicated for the synthesized compounds, from the MIC and MBEC values.

### Symmetry group, molecular size and total energy of the compounds

Density functional theory at M11/ktzvp level of theory was used to optimize geometries of the Mannich benzimidazole compounds (Fig. 3). For each benzimidazole compound the symmetry group was attributed, the length of molecule and total energy were calculated (Table 4). The smallest molecule (8.935 Å) with the highest total energy (− 533.628 Ha), benzimidazole **B**, is one of the most reactive compounds considering its antimicrobial activity. The high stability of a molecule, expressed by a small total energy, of − 1090,231 Ha, in the case of compound **M-3**, is materialized by its low interaction, that is, by a weak antimicrobial activity.

**Table 4:** The symmetry group, length of molecule and total energy of the benzimidazoles

Compound	Symmetry	Length (Å)	E_tot_ (Ha)
**B**	C1	*8.935*	− 533.628
**M-1**	C1	11.139	− 878.897
**M-2**	C1	10.823	− 610.780
**M-3**	C1	12.233	− *1090.231*
**M-4**	C1	13.117	− 1063.781
**M-5**	C1	13.362	− 1047.842

### HOMO–LUMO analysis

Important data on electronic structure are obtained studying the molecular orbitals [73]. The highest occupied molecular orbital (E_HOMO_) and lowest unoccupied molecular orbital (E_LUMO_) energy values were determined using DFT at M11/ktzvp level of theory (Table 5) HOMO and LUMO plots of the benzimidazole compounds can be seen in Fig. 5 The HOMO orbital acts as donating electron, and LUMO orbital acts as electron acceptor [76].

**Table 5:** Chemical reactivity indices of the compounds **M-1–M-5** and **B**

Energy (Hartree)	Mannich bases					BIM
	M-1	M-2	M-3	M-4	M-5	B
E_HOMO_	− 0.324	− 0.324	− 0.309	− 0.324	− 0.317	− 0.322
E_LUMO_	0.045	0.043	0.037	− 0.007	0.026	0.048
E_gap_	− 0.369	− 0.367	− 0.346	− *0.317*	− 0.343	− 0.370
η	0.1845	0.183	0.173	0.1585	0.1715	0.185
µ	− 0.1395	− 0.140	− 0.136	− 0.1655	− 0.1455	− 0.137
ω	0.0527	0.0535	0.1069	0.0864	0.0617	0.050

**Fig. 4:**
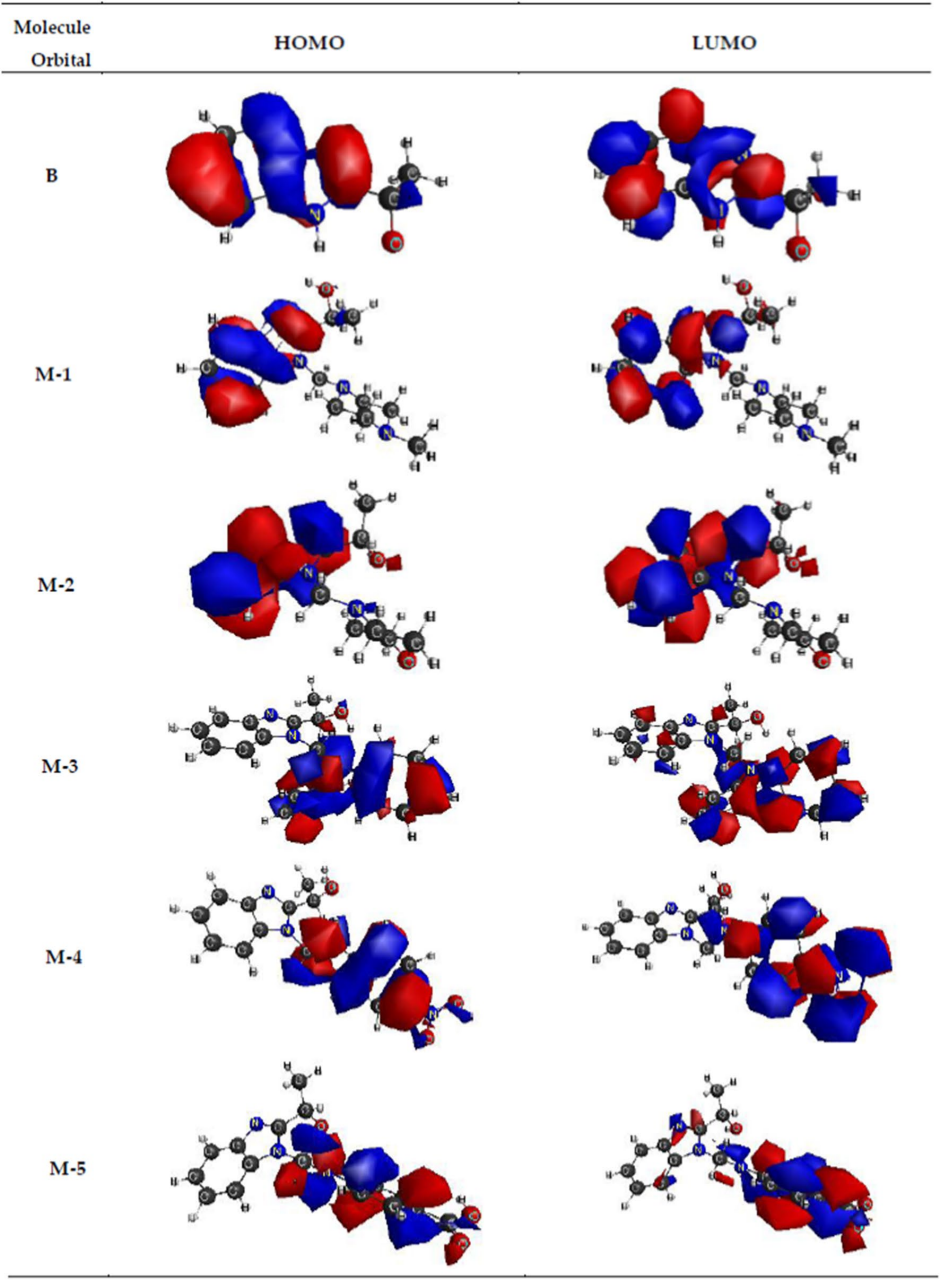
The HOMO–LUMO plots of the benzimidazole compounds **M-1**–**M-5** and **B**

The charge density distribution on the HOMO level is localized on benzimidazole ring in the compounds **B**, **M-1** and **M-2**, meaning on C=C, C–N and C=N bonds, also on the CH (bonded to OH) and oxygen atom; while for the compounds **M-3**, **M-4** and **M-5**, HOMO is localized on the aromatic ring from the amine, on the two phenyl rings, 4-nitroaniline and 4-aminobenzoic acid residues, respectively, nitro and amino groups, nitrogen from amine (N or NH) and also on methylene group (Fig. 4).Charge density on LUMO levels indicates electron density transfer from the amine moiety to benzimidazole ring, to the C–C and C–N bonds. This shifting is very restricted (LUMO plots on benzimidazole are small on **M-3** and **M-5**) because of the two phenyl groups in **M-3** and COOH group in **M-5** with tendency to withdraw electron, a negative inductive effect and will permit a low density charge on benzimidazole ring. In compound **M-4** seems that the charge shifting on benzimidazole ring is not felt at all because of the both inductive and electromer electron attracting effects of the NO_2_ group. This difference in charge distribution will also be reflected in their reactivity.

The energetic values of the HOMO–LUMO levels are at about the same value − 0.324 − 322 Ha and 0.43–0.48 Ha respectively, in the gas phase, for the four compounds **B**, **M-1**, **M-2** and **M-4** except for LUMO of **M-4** with − 0.04 Ha value.

The electronic indices: E_gap_ (E_HOMO_ − E_LUMO_), η (chemical hardness), μ (electronic chemical potential), ω (global electrophilicity index) shown in Table 5 were used to evaluate the chemical reactivity of the benzimidazole compounds.

### Electronic parameters of the benzimidazole compounds

A low HOMO–LUMO gap is reflected in a molecule very reactive and less stable. E_gap_ is the lowest for Mannich base **M-4**, which possessed a good antimicrobial activity, on 50% of all strains tested, for both MIC and MBEC values, but not the best activity of all compounds. A high HOMO–LUMO energy difference is noted for the compound **M-1**, that has the best antimicrobial activity, and also resembling values for the compounds **B** and

**M-2**. Shortly, we expect the decreasing activity in the order **M-4 **>** M-5 **>** M-3 **>** M-2 **>** M-1 **>** B**, but the order is almost totally inverted. Therefore we must consider the contribution of other parameters to biological activity.

Pearson states the best values of the calculated parameters are obtained in case of the minimum HOMO energy values and maximum energy values of LUMO orbitals [77]. That means for compounds **M-1**, **M-2** and **B**, the values of E_gap_ are best fitted with their reactivity and confirm the antimicrobial behaviour of the tested compounds. Moreover, considering HOMO–LUMO energies values, the decreasing order of the biological activity results as follows: **M-1 **>** M-2 **>** B **> **M-4 **>** M-3 **>** M-5**, order that corresponds for the minimum inhibitory concentration variation of the compounds.

Pearson mention that *in nature molecules arranges themselves to be as hard as possible* [77], so the specific parameter hardness η, defined as below, has to be larger, in order to have a molecule biologically reactive. Considering the calculated values of hardness (Table 5), a higher biological activity is expected for compounds **B**, **M-1**, **M-2** and a lower biological activity for **M-3**, **M-4** and **M-5**. In this case, the following order of decreasing biological activity would result **B **>** M-1 **>** M-2 **>** M-3 **>** M-5 **>** M-4**. The Minimum Inhibitory Concentration (MIC) values (Table 2) and the minimum biofilm eradication concentration (MBEC) values (Table 3) show biological activities show biological activities lower than expected for compounds **M-3**, **B** and **M-5**, and higher than expected for **M-4**, because the η values for **M-5**, **M-3** and **B** of 0.1715, 0.173 and 0.185, respectively, are higher than the η value for **M-4** of 0.1585. The better biological activity of **M-4** is related with another parameter, like the charge on atom.

$$\eta = \frac{{E_{LUMO} - E_{HOMO} }}{2}$$

The electronic chemical potential (μ) defines the reactivity of one compound, so a greater value indicates a more reactive compound. So, we will expect the order of decreasing reactivity: **M-3 **> **B **>** M-1 **>** M-2 **>** M-5 **>** M-4**. In this case, a higher activity appears for Mannich base **M-3**, and a lower activity for **M-4**, values that will be explained by the following discussions.

$$\mu = \frac{{E_{HOMO} + E_{LUMO} }}{2}$$

The last parameter, the global electrophilicity index (ω), which assesses the electrophilic nature of one molecule in a relative scale, is best correlated with the biological activities of the benzimidazole compounds, as it was formulated in a previous study [6]. Increasing the global electrophilicity indice is consistent with the decrease in biological activity in the order **M-1 **>** B **>** M-2 **>** M-5 **> **M-4 **>** M-3**. In this case **M-1** appears to be the most biologically active and **M-3** as the least active, which corresponds to the obtained experimental results.

$$\omega = \frac{{\mu^{2} }}{2\eta }$$

## Selected geometrical parameters of the benzimidazole compounds

### Bond lengths

**Fig. 5:**
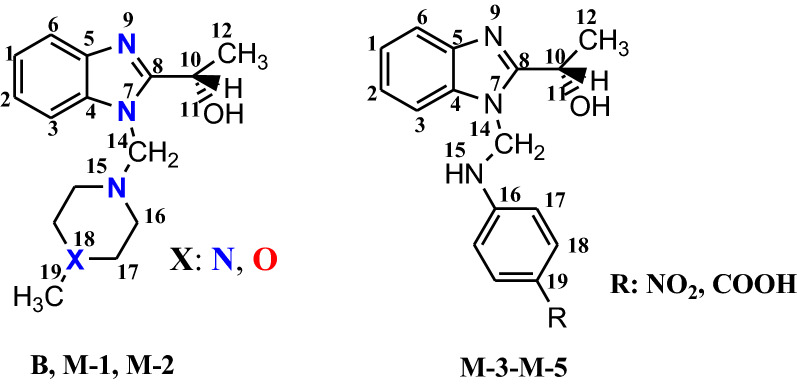
Numbering of the atoms on the studied compounds

Table 6 shows the bond length values of the title benzimidazoles and Fig. 5 indicates the atom numbering in the studied compounds. The resembling values of the bond lengths for the **M-1**, **M-2** and **B** in relation with the same counterparts of the molecules and the same localization of the HOMO–LUMO orbitals may explain the three alike biological activities. Shortening the chemical bond N^15^-C^16^ and enlargement the chemical bond C^14^-N^15^ together with the presence of the frontier orbitals on these atoms could explain the low reactivity of the base **M-3**. The difference between the reactivity of the compounds **M-4** and **M-5** seems to be better related to the charges on the atoms than to the bond lengths that are very similar.

**Table 6:** Bond length values of the discussed benzimidazoles

Chemical bond	Length (Å)					
	M-1	M-2	M-3	M-4	M-5	1B
C^8^-C^10^	1.506	1.509	1.511	1.506	1.505	1.507
C^10^-C^12^	1.528	1.520	1.517	1.517	1.517	1.518
C^10^-O^11^	1.415	1.430	1.426	1.438	1.438	1.427
O^11^-H^20^	0.966	0.963	0.961	0.963	0.963	0.961
N^7^-C^14^	1.445	1.453	1.447	1.442	1.443	–
C^14^-N^15^	1.446	1.441	*1.458*	1.446	1.445	–
N^15^-C^16^	1.458	1.460	*1.429*	1.370	1.374	–
C^16^-C^17^	1.519	1.519	1.397	1.407	1.404	–
C^17^-X^18^	1.454	1.421	1.385	1.373	1.367	–
X^18^-C^19^	1.451	–	1.387	1.388	1.396	–
C^19^-Y^20^	–	–	1.386	1.467	1.493	–
N^21^-O^22^ (NO_2_)	–	–	–	1.213	–	–
C-O (COOH)	–	–	–	–	1.354	–
C = O (COOH)		–	–	–	1.196	–
OH (COOH)		–	–	–	0.961	–

### Bond and dihedral angles

Determination of bond and dihedral angles is very important for the biological activities of the molecules because a molecule with a flat geometry or a symmetric one possess a better antimicrobial activity than a molecule with a distorted geometry [6, 22]. Moreover, the presence of a charge of a heteroatom on a molecule is an advantage for the biological activity. By analyzing the bond and dihedral angles from Table 7, we may conclude: Mannich base **M-1** is composed of two moieties, each of them almost planar, the benzimidazole and 1-methylpiperazine, situated at 111.508 degrees (N^7^C^14^N^15^). The negative charge on the methyl group (− 0.383) seems to increase the biological activity of the compound. Compound **M-2** possessed similar geometry to **M-1**, slightly sharper, but lack of the methyl group leads to a decrease in biological activity, even if the charge of oxygen atom (− 0.301) is present on the aliphatic heterocycle. The perfectly planar geometry of the benzimidazole **B** is reflected in its good reactivity, similar with compound **M-2**.

**Table 7:** Bond and dihedral angles of the benzimidazole compounds

Parameter	Angle value					
	M-1	M-2	M-3	M-4	M-5	1B
C^8^C^10^C^12^	109.668	110.798	110.682	111.723	111.686	110.467
C^8^C^10^O^11^	108.911	108.962	111.489	108.316	108.338	109.028
N^7^C^14^N^15^	*111.508*	*111.566*	112.662	110.528	110.507	*112.093*
N^7^C^8^C^10^C^12^	− 95.951	− 150.864	− 161.515	157.901	− 157.392	77.533
N^9^C^8^C^10^C^12^	*81.831*	34.525	21.463	− 29.587	30.127	*99.982*
N^7^C^8^C^10^O^11^	142.003	86.402	− 40.111	− 29.587	79.257	161.069
N^7^C^14^N^15^C^16^	− 173.275	111.566	− 50.042	− 178.827	177.665	–
C^14^N^15^C^16^C^17^	*177.597*	*177.202*	− 22.117	168.667	− 168.389	–
C^16^C^17^N^18^C^19^	*177.057*	–	–	–	–	–
C^17^C^16^N^15^C^21^	–		− 20.795	–	–	
C^18^C^19^NO	–	–	–	*− 179.820*	–	–
C^18^C^19^CO	–	–	–	–	20.368	–

The compound **M-3** has a very twisted geometry, three very sharp dihedral angles: 21.463 (N^9^C^8^C^10^C^12^), − 22.117 (C^14^N^15^C^16^C^17^) and − 20.795 (C^17^C^16^N^15^C^21^), resulting in a drastic decrease in antimicrobial activity. The geometries of **M-4** and **M-5** are somewhat similar. Compound **M-4** is composed of two planar moieties, which greatly contribute to the increase of antimicrobial activity, while the 4-aminobenzoic acid residue has a 20.368 (dihedral angle C^18^C^19^CO) degree deviation from the planarity, which leads to a large decrease in biological activity.

### Mulliken atomic charges

Table 8 presents the Mulliken atomic charges calculated using DFT at M11/ktzvp level of theory. Previous studies showed that an increased charge favors a good biological activity [6]. It can be said this observation is valid for **M-1**, **M-2** and **M-4** bases due to the two charges localized on the following atoms: on nitrogen (N-18) of − 0.255, on oxygen (O-18) of − 0.301, and on nitrogen (from NO_2_) of 0.317, and also for **B** because of the charge on O-11 of − 0.435.

**Table 8:** Mulliken atomic charges of compounds **M-1**–**M-5** and **B**

Atom	Mulliken charge					
	M-1	M-2	M-3	M-4	M-5	B^a^
C-1 (Benzimidazole)	− 0.149	− 0.150	− 0.135	− 0.132	− 0.132	− 0.132
C-2 (Benzimidazole)	− 0.182	− 0.182	− 0.168	− 0.192	− 0.198	− 0.189
C-3 (Benzimidazole)	− 0.058	− 0.057	− 0.105	− 0.057	− 0.056	− 0.089
C-4 (Benzimidazole)	− 0.042	− 0.037	− 0.067	− 0.041	− 0.042	0.013
C-5(Benzimidazole)	0.009	0.012	0.028	0.022	0.023	0.010
C-6 (Benzimidazole)	− 0.113	− 0.122	− 0.136	− 0.129	− 0.130	− 0.143
N-7 (Benzimidazole)	− 0.296	− 0.321	− 0.301	− 0.349	− 0.347	− 0.389
C-8 (Benzimidazole)	0.194	− 0.186	0.321	0.249	0.247	0.167
N-9 (Benzimidazole)	− 0.386	− 0.317	− 0.320	− 0.315	− 0.316	− 0.312
C-10	− 0.016	− 0.028	− 0.149	− 0.196	− 0.195	− 0.020
O-11	− ***0.432***	− ***0.443***	− ***0.447***	− ***0.451***	− ***0.450***	− ***0.435***
C-12	− 0.408	− 0.466	− 0.471	− 0.467	− 0.466	− 0.349
C-14	− 0.319	− 0.186	− 0.251	− 0.136	− 0.140	–
N-15	− 0.241	− 0.323	− ***0.527***	− 0.488	− ***0.491***	–
C-16	− 0.262	− 0.230	− 0.178	0.253	0.256	–
C-17	− 0.350	− 0.215	− 0.106	− 0.225	− 0.280	–
X-18	− ***0.255*** (N)	− ***0.301***(O)	− 0.172	− 0.099	− 0.027	–
C-19 (CH_3_)	− 0.383	–	− 0.128	− 0.143	− 0.441	–
N-20 (from NO_2_)	–	–	–	***0.317***	–	–
O-21 (from NO_2_, COOH)	–	–	–	− *0.288*	− 0.384	–
O-22 (from NO_2_, COOH)	–	–	–	− *0.286*	− 0.345	–
C-20 (from COOH)	–	–	–	–	0.502	–
H-22 (from COOH)	–	–	–	–	0.306	–

^a^From Ref. [6]

Only a high charge on the O-11 atom is not sufficient for good antimicrobial activity, as shown in Table 8. It seems that a too high charge on the amine nitrogen (N-15) leads to a decrease in biological activity, as can be seen for bases **M-3** and **M-5** with charges of − 0.527 and − 0.491, respectively. The high atomic charge on some carbon atoms (C-12, C-14, C-16, C-17, C-19) is explained by the neighborhood of one or more electronegative elements and the rearrangement of the charge. In this case, the negative charge on the carbon can be considered as a formal charge.

It is obvious that the **B** and **M-1** molecules stand out, with an antimicrobial activity superior to the other compounds due to their structural characteristics, which constitute advantages for the structure–activity relationship, among which we can mention:

- small size of molecules, with a length of 8935 Å and 11,139 Å respectively;
- almost identical HOMO–LUMO energy difference of the two compounds (− 0.370 and − 0.369 Ha respectively);
- global electrophilicity indices have the lowest values of all (0.050 and 0.0527 respectively);
- the almost flat structure of the two molecules, which results from the values of the angles presented in Table 6, constitutes a major advantage over the other molecules that have a three-dimensional and even distorted structure in some cases (**M-3**);
- a minimum Mulliken charge on oxygen atom (from OH group), which means a higher hydrophobicity, is materialized by an increase in antimicrobial activity;
- the presence of the two nitrogen heteroatoms in the piperazine heterocycle, as well as the methyl group linked to the nitrogen atom, favors a higher total Mulliken charge, fact materialized by better biological properties than the other homologous Mannich bases;
- the majority localization of HOMO and LUMO orbitals on the benzimidazole heterocycle in molecules **B** and **M-1**, as well as the very close energy values of these orbitals (Table 4), are advantages that lead to a better antimicrobial activity.

### Experimental section

Elemental analysis was performed with a “multi EA 4000” device from “Analytik Jena”. Fourier transform infrared (FTIR) spectra have been acquired by using a “Vertex 70-Bruker” spectrophotometer, in KBr pellets. The NMR spectra were recorded on Bruker Advance Ultrashield Plus spectrometer operating at 300.18 MHz for ^1^H and 125 MHz for ^13^C. The enantiomeric ratios were determined by HPLC analysis with a CHIRALPAK AS column (4.6 × 250 mm), [254 nm, 0.50 mL/minute, i-propanol-hexane (90:10)]. Optical rotations were recorded on a Perkin-Elmer 236 polarimeter and the [α]_D_ values are given in units of 10^−1^ deg cm^2^ g^−1^.Melting points were determined in open capillary tubes using a STUART SMP3 electric melting point apparatus and are uncorrected. M + 1 peaks were determined on an Agilent 1100 series and an Agilent Ion Trap SL mass spectrometer (Santa Clara, CA, USA), operating at 70 eV.

#### Synthesis of (S)-1-(1H-Benzo[d]imidazole-2-yl)ethanol (B)

A mixture of (S)-2-hydroxypropanoic acid (50 mmol), *o*-phenylenediamine (50 mmol) and 4 N hydrochloric acid, thoroughly grounded with a pestle in a mortar at room temperature until liquefied, was subsequently heated at 140 °C for 2 h. The progress of the reaction was monitored by thin-layer chromatography (TLC). After cooling, the resulting mass was washed with ammonia, filtered, and the final product was recrystallized from ethanol. Colourless solid. Yield 80% (3.64 g) m.p. 177–178 °C; similar to [29] (m.p. 176–178 °C). IR (cm^−1^, KBr): 3356 (aromatic -NH bending), 3364 (OH stretching), 3070 (aromatic C-H stretching), 1505 (C–N stretching), 1458 (–C=C stretching), 1315 (–C–N stretching); ^1^H-NMR, (300 MHz, DMSO-*d*_6_): δ = 12.23 (s, 1H, N*H*), 7.54 (d, H-6, ^3^J = 8.6 Hz), 7.44 (d, H-3, ^3^J = 8.6 Hz), 7.12 (d, 2H, H-1,2, ^3^J = 5.7 Hz), 5.77 (t, 1H, O*H*, ^3^J = 4.5 Hz), 4.94 (q, 1H, C*H*, ^3^J = 6.5 Hz), 1.51 (d, 3H, C*H*_3_, ^3^J = 6.6 Hz) ppm. ^13^C-NMR, (125 MHz, DMSO-*d*_6_): δ = 158.6 (C-8), 143.1 (C-4), 134.1 (C-5), 121.6 and 120.9 (C-1 and C-2), 118.4 (C-3), 111.3 (C-6), 63.7 (*C*H), 22.9 (*C*H_3_) ppm. Elemental analysis (%) found for C_9_H_10_N_2_O: C, 66.57; H, 6.25; N, 17.29; O, 9.89. calcd.: C, 66.65; H, 6.21; N, 17.27; O, 9.86%. MS (ESI, 70 eV): m/z calc. for C_9_H_10_N_2_O [M+H]^+^: 163.19, found 163.18.

#### Synthesis of the Mannich bases M-1–M-5

A mixture of (S)-1-(1*H*-benzo[d]imidazole-2-yl)ethanol (**B**) (10 mmol), formaldehyde (10 mmol), the corresponding amine (1-methylpiperazine, morpholine, diphenylamine, 4-nitroaniline, 4-amino benzoic acid) (10 mmol) and 4–5 drops of concentrated hydrochloric acid in ethanol was stirred at room temperature for 2 h and then refluxed for additional 3 h. After cooling, Mannich bases were filtered under suction, dried, and re-crystallized from dimethylformamide (DMF).

##### (*S*)-1-(1-((4-Methylpiperazin-1-yl)methyl)-1H-benzo[d]imidazole-2-yl)ethanol (M-1)

White solid. Yield 73% (2 g). m.p. 153–154 °C. *R*_*f*_= 0.72 (silica, EtOAc). IR (cm^−1^, KBr): 3350 (OH stretching), 2977, 2939 (CH arene stretching), 1459 (CH_2_ bending), 1384 (C–N stretching), 1370 (aromatic tertiary amine), 1162 (C–N stretching tertiary amine), 819 (out-of-plane CH bending), 745 (*o*-phenylene). ^1^H-NMR, (300 MHz, DMSO-*d*_6_): δ = 7.62 (d, 2H, H-3,6, ^3^J = 8.6 Hz), 7.19 (d, 2H, H-1,2, ^3^J = 5.7 Hz), 5.79 (brs, 1H, O*H*), 5.10 (dd, 2H, C*H*_2_, ^3^J = 6.5 Hz) 4.94 (q, 1H, C*H*, ^3^J = 6.5 Hz), 2.5 (d, 8H, 4 C*H*_2_, ^3^J = 6.5 Hz), 2.12 (d, 3H, C*H*_3_, ^3^J = 6.7 Hz), 1.50 (d, 3H, C*H*_3_, ^3^J = 6.6 Hz) ppm. ^13^C-NMR, (125 MHz, DMSO-*d*_6_): δ = 157.12, 141.40, 136.12, 122.37, 121.52, 119.10, 110.73, 64.04, 61.40, 54.34, 49.80, 45.63, 21.05 ppm. Elemental analysis (%) found for C_15_H_22_N_4_O: C, 65.59; H, 8.13; N, 20.47; O, 5.71. calcd.: C, 65.66; H, 8.08; N, 20.42; O, 5.83%. MS (ESI, 70 eV): m/z calc. for C_15_H_22_N_4_O [M+H]^+^: 275.37, found 275.39.

##### (*S*)-1-(1-((4-Morpholin-1-yl)methyl)-1H-benzo[d]imidazole-2-yl)ethanol (M-2)

White solid. Yield 75% (1.95 g). m.p. 137–138 °C. *R*_*f*_= 0.56 (silica, EtOAc). IR (cm^−1^, KBr): 3350 ((OH stretching), 3056 (CH arene stretching), 1457 (CH_2_ bending), 1317, 1308, 1272 (C–N stretching), 1105 (C–O–C cyclic ether stretching), 808 (out-of-plane CH bending), 742 (*o*-phenylene). ^1^H-NMR, (300 MHz, DMSO-*d*_6_): δ = 7.62 (d, H-6, ^3^J = 8.6 Hz), 7.48 (s, H-3), 7.15 (d, 2H, H-1,2, ^3^J = 5.7 Hz), 5.74 (s, 1H, O*H*), 5.12 (dd, 2H, C*H*_2_, ^3^J = 6.5 Hz), 4.96 (d, 1H, C*H*, ^3^J = 6.5 Hz), 3.53 (d, 2H, C*H*_2_, ^3^J = 6.5 Hz), 3.36 (d, 2H, C*H*_2_, ^3^J = 6.5 Hz), 2.50 (4H, 2 C*H*_2_, ^3^J = 6.5 Hz), 1.51 (d, 3H, C*H*_3_, ^3^J = 6.6 Hz) ppm. ^13^C-NMR, (125 MHz, DMSO-*d*_6_): δ = 157.06, 141.44, 136.11, 122.35, 121.54, 121.21, 119.06, 110.85, 66.16, 65.96, 63.67, 61.45, 50.40, 22.97 ppm. Elemental analysis (%) found for C_14_H_19_N_3_O_2_: C, 64.39; H, 7.27; N, 16.14; O, 5.71. calcd.: C, 64.35; H, 7.32; N, 16.08; O, 12.19%. MS (ESI, 70 eV): m/z calc. for C_14_H_19_N_3_O_2_ [M+H]^+^: 262.32, found 262.37.

##### (*S*)-1-(1-((Diphenylamino)methyl)-1H-benzo[d]imidazole-2-yl)ethanol (M-3)

Light-yellow solid. Yield 81% (2.78 g). m.p. 168–169 °C. *R*_*f*_= 0.54 (silica, EtOAc). IR (cm^−1^, KBr): 3450 (OH stretching), 3085, 3055 (CH arene stretching), 1621 (C=C stretching), 1495 (CH_2_ bending), 1316, 1308, 1272 (C–N stretching), 1104 (C–C–C bending), 744 (*o*-phenylene). ^1^H-NMR, (300 MHz, DMSO-*d*_6_): δ = 7.74 (d, H-6, ^3^J = 8.6 Hz), 7.53 (d, H-3, ^3^J = 8.6 Hz), 7.44 (m, 4H, C*H* aromatic), 7.09 (m, 6H, C*H* aromatic), 6.91 (d, 1H, ^3^J = 5.7 Hz), 6.84 (d, 1H, ^3^J = 5.7 Hz), 6.01 (dd, 2H, C*H*_2_, ^3^J = 6.5 Hz), 5.74 (s, 1H, O*H*), 4.72 (d, 1H, C*H*, ^3^J = 6.5 Hz), 1.60 (d, 3H, C*H*_3_, ^3^J = 6.6 Hz) ppm. ^13^C-NMR, (125 MHz, DMSO-*d*_6_): δ = 158.67, 147.71, 143.47, 129.18, 127.52, 126.63, 124.64, 121.29, 121.10, 120.40, 119.68, 117.05, 116.76, 116.46, 81.27, 63.67, 23.05 ppm. Elemental analysis (%) found for C_22_H_21_N_3_O: C, 76.99; H, 6.22; N, 12.08; O, 4.71. calcd.: C, 76.94; H, 6.16; N, 12.23; O, 4.66%. MS (ESI, 70 eV): m/z calc. for C_22_H_21_N_3_O [M+H]^+^: 343.43, found 343.35.

##### (*S*)-1-(1-((4-nitrophenylamino)methyl)-1H-benzo[d]imidazole-2-yl)ethanol (M-4)

Yellow solid. Yield 83% (2.78 g). m.p. 193–194 °C. *R*_*f*_= 0.57 (silica, EtOAc). IR (cm^−1^, KBr): 3469 (OH stretching), 1603 (NH stretching), 1530 (CH_2_ bending), 1498, 1470 (aromatic nitro), 1321, 1299, 1268 (C–N stretching), 1189 (C–N tertiary amine), 1109, 1097 (C–C–C bending), 835 (NH stretching), 753 (*o*-phenylene).

^1^H-NMR, (300 MHz, DMSO-*d*_6_): δ = 8.02 (d, 2H, ^3^J = 8.6 Hz), 7.92 (s, N*H*), 7.63 (d, 1H, ^3^J = 5.7 Hz), 7.46 (d, 1H, ^3^J = 5.7 Hz), 7.09 (d, 2H, ^3^J = 8.6 Hz), 6.80 (d, 2H, ^3^J = 8.6 Hz), 5.72 (s, 1H, O*H*), 5.46 (d, 2H, C*H*_2_, ^3^J = 6.5 Hz), 4.92 (d, 1H, C*H*, ^3^J = 6.5 Hz), 1.51 (d, 3H, C*H*_3_, ^3^J = 6.6 Hz) ppm. ^13^C-NMR, (125 MHz, DMSO-*d*_6_): δ = 158.54, 153.23, 138.56, 136.79, 134.38, 126.04, 125.96, 125.52, 125.27, 125.10, 121.53, 120.84, 113.56, 81.38, 63.39, 22.96 ppm. Elemental analysis (%) found for C_16_H_16_N_4_O_3_: C, 61.59; H, 5.22; N, 17.98; O, 15.20. calcd.: C, 61.53; H, 5.16; N, 17.93; O, 15.37%. MS (ESI, 70 eV): m/z calc. for C_16_H_16_N_4_O_3_ [M+H]^+^: 313.34, found 313.38.

##### (*S*)-4-((2-(1-Hydroxyethyl)-1H-benzo[d]imidazol-1-yl)methylamino)benzoic acid (M-5)

Yellow solid. Yield 78% (2.42 g). m.p. 223–224 °C. *R*_*f*_= 0.49 (silica, EtOAc). IR (cm^−1^, KBr): 3400–3100 (OH stretching), 2925 (CH arene stretching), 1682 (C=C stretching), 1603 (NH stretching), 1519 (CH_2_ bending), 1315, 1241 (C–N stretching), 1175 (C–N tertiary amine), 1084 (C–C–C bending), 841 (NH stretching), 745, 699 (*o*-phenylene). ^1^H-NMR, (300 MHz, DMSO-*d*_6_): δ = 7.72 (d, 2H, ^3^J = 8.6 Hz), 7.61 (d, 2H, ^3^J = 5.7 Hz), 6.88 (d, 2H, ^3^J = 8.6 Hz), 6.71 (d, 1H, ^3^J = 8.6 Hz), 6.58 (d, 1H, ^3^J = 8.6 Hz), 5.79 (dd, 2H, C*H*_2_, ^3^J = 6.5 Hz), 5.34 (brs, 1H, O*H*), 4.94 (q, 1H, C*H*, ^3^J = 6.5 Hz), 1.51 (d, 3H, C*H*_3_, ^3^J = 6.6 Hz) ppm. ^13^C-NMR, (125 MHz, DMSO-*d*_6_): δ = 167.47, 158.57, 156.28, 151.32, 148.90, 140.82, 135.76, 131.22, 130.57, 121.40, 119.36, 117.83, 115.94, 109.95, 83.77, 72.19, 22.97 ppm. Elemental analysis (%) found for C_17_H_17_N_3_O_3_: C, 65.55; H, 5.55; N, 13.54; O, 15.35. calcd.: C, 65.58; H, 5.50; N, 13.49; O, 15.42%. MS (ESI, 70 eV): m/z calc. for C_17_H_17_N_3_O_3_ [M+H]^+^: 313.34, found 313.38.

## Materials and methods

### Chemicals

All reagents and solvents (provided by Sigma-Aldrich) were used without any further purification.

### Evaluation of the antimicrobial activity

#### Qualitative screening of the antimicrobial activity

Standardized bacterial suspensions with a density of 1.5–3 × 10^8^ CFU mL^−1^ (corresponding to the 0.5 *McFarland* nephelometric standard) were obtained from 15 to 18 h fresh bacterial cultures developed on solid media. The compounds were suspended in DMSO to prepare a stock solution of 10 mg mL^−1^ concentration. The antimicrobial activity was tested on *Mueller*–*Hinton Agar* (MHA) medium. The qualitative screening was performed by an adapted disc diffusion method [60, 78].

#### Quantitative assay of the antimicrobial activity

The quantitative assay of the antimicrobial activity was performed by liquid medium microdilution method in 96 multi-well plates. Two-fold serial dilutions of the compounds solutions (ranging between 1000 mg and 4 mg mL^−1^) were performed in a 200 mL volume of broth, and each was well seeded with 50 mL microbial inoculum. Bacterial culture positive controls (wells containing culture medium seeded with the microbial inoculum) as well negative sterility controls (containing only culture medium) were used. The influence of the DMSO solvent was also quantified in a series of wells containing DMSO, diluted accordingly with the dilution scheme used for the tested compounds. The multi-well plates were incubated for 24 h at 37 °C, and the minimal inhibitory concentration (MIC) values were considered as the lowest concentration of the tested compound that inhibited the visible growth of the microbial overnight cultures, as compared to the positive control, correlated with a decreased value of the absorbance read at 600 nm (by using an “Apollo LB 911” ELISA reader). All trials were performed in triplicate and the results were stated as mean.

#### Quantitative assay of the anti-biofilm activity

In order to evaluate the influence of the obtained compounds upon the colonization ability of microbial strains to the inert substratum, a microtiter method was employed. The multi-well plates used for the MIC assay were emptied and washed three times with phosphate buffered saline. The biofilm formed on the plastic wells wall. After it was fixed for 5 min with cold methanol and coloured for 15 min by violet crystal solution, it was re-suspended in a 33% acetic acid solution. The minimal biofilm eradication concentration (MBEC) values were considered at the lowest concentration of the tested compound that inhibited the development of biofilm on the plate wells, as revealed by the decreased values of the optical density of the coloured solution at 490 nm, and as compared to that of the positive control [72]. All trials were performed in triplicate and the results were stated as mean.

### Computational and modeling details

A quantum mechanical modeling method was implemented for each benzimidazole compound, using the GAMESS 2012 software [79], in order to assess their structural parameters. The modeling was performed on a computer cluster consisting of 12 nodes and 96 cores running on Linux CentOS. The results were visualized using wxMacMolPlt [80]. The molecular geometries of the benzimidazoles were optimized by using DFT at M11/ktzvp level of theory. Truhlar’s M11 [81] is a modern range-separated hybrid functional that provides better results compared to the traditional B3LYP functional class of approximations to the exchange correlation energy in DFT. Also, the basis set that we used is a more recent one (Karlsruhe valence triple zeta basis with a set of single polarization), introduced by Prof. Ahlrichs [82, 83]. The parameters used for geometry optimization are the default ones used in GAMESS, and the geometry used during the calculations is described by “natural internal coordinates” generated by the software.

## Conclusions

We reported here the synthesis and the characterization of a series of new chiral Mannich benzimidazole bases starting from (S)-1-(1*H*-benzo[d]imidazole-2-yl)ethanol (**B**). The analysis of their antimicrobial activity allowed us to determine that Mannich base **M-1** shows the best antimicrobial activity expressed both as MIC value and as anti-biofilm activity. For all new compounds a better antifungal activity than the antimicrobial activity was reported. For all compounds, the calculated chemical reactivity indices were correlated with their antimicrobial behaviour. Molecules **B** and **M-1** have superior antimicrobial activity, due to their structural characteristics, like: almost flat structure of the two molecules, higher hydrophobicities, minimum Mulliken charges on oxygen atom (from OH group) and lowest values of global electrophilicity indices (of 0.050 and 0.0527, respectively). The compound **M-4** shows the best values for the anti-biofilm activity, of 0.030 μg mL^−1^ against *Staphylococcus aureus* and *Candida albicans*. The similar reactivities of the three compounds **M-1**, **M-2** and **B** was related mainly with localization of HOMO–LUMO orbitals and with tridimensional structures of the molecules. The flatness of the molecule is an advantage for a good biological activity, as can be seen from the optimized geometries of the studied compounds, **M-2** and **B**. Similarly, the increased charges on heteroatoms in molecules **M-1**, **M-2** and **M-4**, contribute to a better antimicrobial activity.

## Supplementary information

**Additional file 1: Fig. S1.** Comparison of experimental and simulated IR spectra of the benzimidazole compounds: (a) Experimental FTIR spectra; (b) Simulated IR spectra. **Fig. S2.** FTIR spectra of Mannich base **M-1**. **Fig. S3.** FTIR spectra of Mannich base **M-2**. **Fig. S4.** FTIR spectra of Mannich base **M-3**. **Fig. S5.** FTIR spectra of Mannich base **M-4**. **Fig. S6.** FTIR spectra of Mannich base **M-5**. **Table S1.** Important infrared bands (in cm^−1^) of the benzimidazoles: **B**, **M-1**–**M-5** (experimental and calculated). **Fig. S7.**^1^H NMR spectra of Mannich base **M-1**. **Fig. S8.**^13^C NMR spectra of Mannich base **M-1**. **Fig. S9.**^1^H NMR spectra of Mannich base **M-2**. **Fig. S10.**^13^C NMR spectra of Mannich base **M-2**. **Fig. S11.**^1^H NMR spectra of Mannich base **M-3**. **Fig. S12.**^13^C NMR spectra of Mannich base **M-3**. **Fig. S13.**^1^H NMR spectra of Mannich base **M-4**. **Fig. S13.**^1^H NMR spectra of Mannich base **M-4**. **Fig. S14.**^13^C NMR spectra of Mannich base **M-4**. **Fig. S15.**^1^H NMR spectra of Mannich base **M-5**. **Fig. S16.** 13C NMR spectra of Mannich base **M-5**.

## Data Availability

The datasets and samples of the compounds used during the current study are available from the corresponding author on reasonable request.

## Abbreviations

DFT: Density functional theory
NMR: Nuclear magnetic resonance
FTIR: Fourrier transform infra-red
HPLC: High-performance liquid chromatography
HOMO: The highest occupied molecular orbital
LUMO: The lowest unoccupied molecular orbital
MIC: Minimum inhibitory concentration
MBEC: Minimum biofilm eradication concentration
MBSC: Multiband superconducting model
