# Supplementary material for: Synthesis, density functional theory study and in vitro antimicrobial evaluation of new benzimidazole Mannich bases
Source: BMC Chem. 2020 Jul 25;14(1):45. doi: 10.1186/s13065-020-00697-z (PMC7382033; doi:10.1186/s13065-020-00697-z)
Supplement: Supplementary file 1 — Additional file 1: Fig. S1. Comparison of experimental and simulated IR spectra of the benzimidazole compounds: (a) Experimental FTIR spectra; (b) Simulated IR spectra. Fig. S2. FTIR spectra of Mannich base M-1. Fig. S3. FTIR spectra of Mannich base M-2. Fig. S4. FTIR spectra of Mannich base M-3. Fig. S5. FTIR spectra of Mannich base M-4. Fig. S6. FTIR spectra of Mannich base M-5. Table S1. Important infrared bands (in cm−1) of the benzimidazoles: B, M-1–M-5 (experimental and calculated). Fig. S7.1H NMR spectra of Mannich base M-1. Fig. S8.13C NMR spectra of Mannich base M-1. Fig. S9.1H NMR spectra of Mannich base M-2. Fig. S10.13C NMR spectra of Mannich base M-2. Fig. S11.1H NMR spectra of Mannich base M-3. Fig. S12.13C NMR spectra of Mannich base M-3. Fig. S13.1H NMR spectra of Mannich base M-4. Fig. S13.1H NMR spectra of Mannich base M-4. Fig. S14.13C NMR spectra of Mannich base M-4. Fig. S15.1H NMR spectra of Mannich base M-5. Fig. S16. 13C NMR spectra of Mannich base M-5. [file 13065_2020_697_MOESM1_ESM.doc]

**Addition file 1**

Synthesis, DFT study and *in vitro* antimicrobial evaluation of new benzimidazole Mannich bases

Maria Marinescu ^1^,*, Ludmila Otilia Cinteza ^2^,*, George Iuliu Marton ^3^, Mariana-Carmen Chifiriuc ^4,5^, Marcela Popa ^4,5^, Ioana Stanculescu ^2^, Christina Zalaru ^1^, Cristina-Elena Stavarache ^6^

^1^ Department of Organic Chemistry, Biochemistry and Catalysis, Faculty of Chemistry, University of Bucharest; Bucharest 050663, Romania.

^2^ Department of Physical Chemistry, Faculty of Chemistry, University of Bucharest; Bucharest 030018, Romania,

^3^ University “Politehnica” of Bucharest, Faculty of Applied Chemistry and Materials Science, 1-7 Polizu, 011061, Bucharest, Romania

^4^ Department of Botanic-Microbiology, Faculty of Biology, University of Bucharest, 1-3 Aleea Portocalilor, 60101, Bucharest, Romania

^5^ Research Institute of the University of Bucharest, 91-95 Splaiul Independentei, 050095, Bucharest, Romania

^6^ Institute of Organic Chemistry “C.D. Nenitzescu” of the Romanian Academy, 202B Splaiul Independentei, 060023, Bucharest, Romania

***** Correspondence: [maria.marinescu@chimie.unibuc.ro](mailto:maria.marinescu@chimie.unibuc.ro); [maria7marinescu@yahoo.com](mailto:maria7marinescu@yahoo.com).;

[ocinteza@gw-chimie.math.unibuc.ro](mailto:ocinteza@gw-chimie.math.unibuc.ro); Tel.: +40-214-120-1400

1. **Fig. S1.** Comparison of experimental and simulated IR spectra of the benzimidazole compounds: (a) Experimental FTIR spectra; (b) Simulated IR spectra.

2. The *FTIR spectra* of the compounds **M-1 — M-5** …………………………………………………….… **Fig**.**S2 – Fig.S6**

3. **Table S1** Important infrared bands (in cm^-1^) of the benzimidazole compounds: **B**, **M-1**-**M-5** (experimental and calculated)

4. The *NMR spectra* for compounds **M-1 — M-5** …………………………… …………………….… **Fig.S7 – Fig.S16**

**
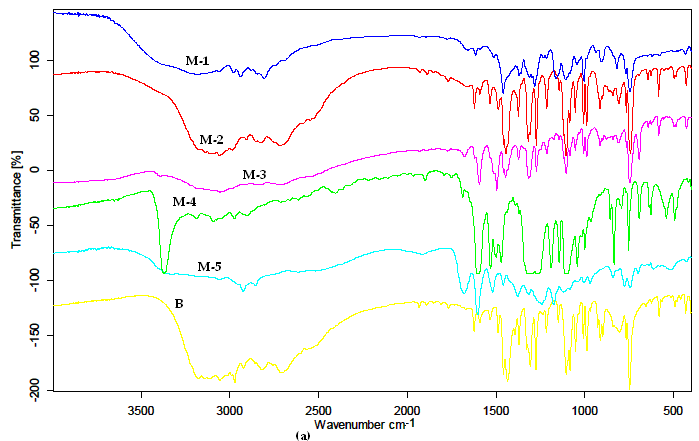
**


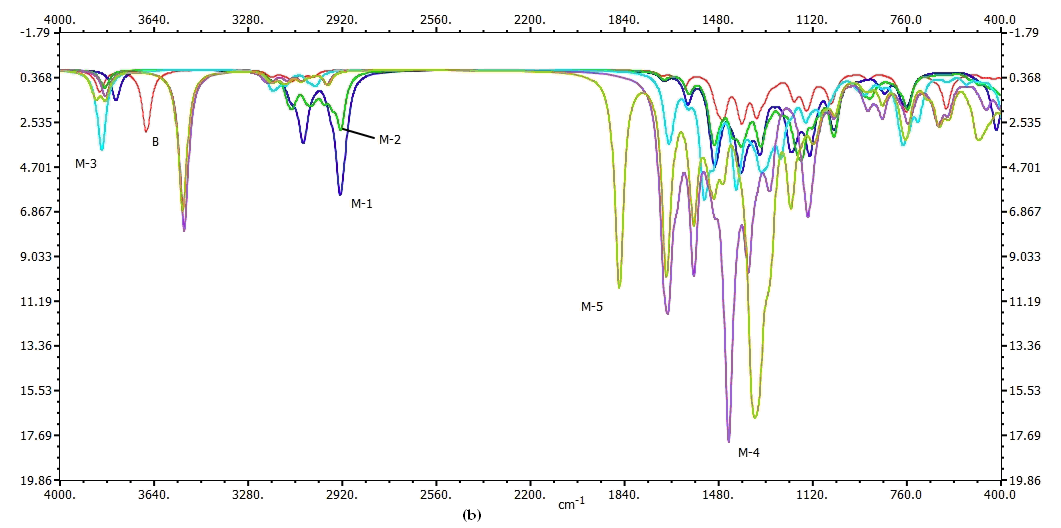


**Fig. S1.** Comparison of experimental and simulated IR spectra of the benzimidazole compounds: (a) Experimental FTIR spectra; (b) Simulated IR spectra.

**Addition file 1: Fig. S1.** Comparison of experimental and simulated IR spectra of the benzimidazole compounds: (a) Experimental FTIR spectra; (b) Simulated IR spectra. **Fig.** **S2.** FTIR spectra of Mannich base **M-1**. **Fig. S3.** FTIR spectra of Mannich base **M-2**. **Fig. S4.** FTIR spectra of Mannich base **M-3**. **Fig. S5.** FTIR spectra of Mannich base **M-4**. **Fig. S6.** FTIR spectra of Mannich base **M-5**. **Table S1** Important infrared bands (in cm^-1^) of the benzimidazole compounds: **B**, **M-1**-**M-5** (experimental and calculated). **Fig. S7.** ^1^H NMR spectra of Mannich base **M-1**. **Fig. S8.** ^13^C NMR spectra of Mannich base **M-1**. **Fig.S9.** ^1^H NMR spectra of Mannich base **M-2**. **Fig.S10.** ^13^C NMR spectra of Mannich base **M-2**. **Fig.S11.** ^1^H NMR spectra of Mannich base **M-3**. **Fig.S12.** ^13^C NMR spectra of Mannich base **M-3**. **Fig.S13.** ^1^H NMR spectra of Mannich base **M-4**. **Fig.S13.** ^1^H NMR spectra of Mannich base **M-4**. **Fig.S14.** ^13^C NMR spectra of Mannich base **M-4**. **Fig.S15.** ^1^H NMR spectra of Mannich base **M-5**. **Fig.S16.** ^13^C NMR spectra of Mannich base **M-5**.

**1. The FTIR spectra**

**Fig.** **S2.** FTIR spectra of Mannich base **M-1**.

**Fig. S3.** FTIR spectra of Mannich base **M-2**.

**Fig. S4.** FTIR spectra of Mannich base **M-3**.

**Fig. S5.** FTIR spectra of Mannich base **M-4**.

**Fig. S6.** FTIR spectra of Mannich base **M-5**.

**Table S1** Important infrared bands (in cm^-1^) of the benzimidazole compounds: **B**, **M-1**-**M-5** (experimental and calculated)

| Assignment | Compound | | | | | |
| --- | --- | --- | --- | --- | --- | --- |
|  | **B** | **M-1** | **M-2** | **M-3** | **M-4** | **M-5** |
| ν(OH) _exp_ | 3100-3200 | 3210 | 3210 | 3300 | 3409 | 3050-3450 |
| ν(OH) _calc_ | 3640 | 3500-3600 | 3600 | 3250 | 3500 | 3500 |
| ν(COOH) _exp_ | - | - | - | - | - | 3100-3400 |
| ν(COOH) _calc_ | - | - | - | - | - | 3200 |
| ν(CH)_arene exp_ | 3051 | 3057 | 3056 | 3055 | 3051 | 3057 |
| ν(CH) _arene calc_ | 3025 | 3090 | 3085 | 3050 | 3056 | 3060 |
| ν(C=C)_stretch exp_ | - | - | - | 1620 | - | 1682 |
| ν(C=C)_stretch calc_ | - | - | - | 1625 | - | 1720 |
| ν(NH)_stretch exp_ | - | - | - | - | 1603 | 1603 |
| ν(NH)_stretch calc_ | - | - | - | - | 1610 | 1615 |
| δ(CH_2_)_bending exp_ | 1460 | 1459 | 1457 | 1495 | 1530 | 1519 |
| δ(CH_2_)_bending calc_ | 1480 | 1485 | 1483 | 1500 | 1580 | 1560 |
| ν(NO_2_)_stretch exp_ | - | - | - | - | 1498, 1470, 1321 | - |
| ν(NO_2_)_stretch calc_ | - | - | - | - | 1480 | - |
| ν(C–N) _strech exp_ | - | 1370 | 1308, 1272 | - | 1321, 1299, 1268 | 1377, 1316 |
| ν(C–N) _strech_  calc | - | 1395 | 1390 | - | 1330 | 1380 |
| ν_(C-N) tertiary amine exp_ | - | 1311 | 1317 | 1316 | 1316 | 1315 |
| ν(C-N) tertiary amine calc | - | 1336 | 1343 | 1320 | 1320 | 1335 |
| δ(C-O-C)_cyclic ether exp_ | - | - | 1105 | - | - | - |
| δ(C-O-C)_cyclic ether calc_ | - | - | 1125 | - | - | - |
| δ(C-C-C)_bending exp_ | - | - | - | 1104 | 1108 | 1175 |
| δ(C-C-C)_bending calc_ | - | - | - | 1107 | 1120 | 1210 |
| δ_(NH)strech exp_ | - | - | - | - | 834 | 841 |
| δ_(NH)strech calc_ | - | - | - | - | 854 | 856 |
| δ_C-H arom out of plane exp_ | 820 | 819 | 808 | - | - | - |
| δ_C-H arom out of plane calc_ | 845 | 851 | 853 | - | - | - |
| δ_(o-Phenylene) exp_ | 745 | 745 | 742 | 744 | 753 | 745, 700 |
| δ_(o-Phenylene) calc_ | 760 | 764 | 765 | 765 | 760 | 763 |

**2. The ^1^H and the ^13^C-NMR spectra**


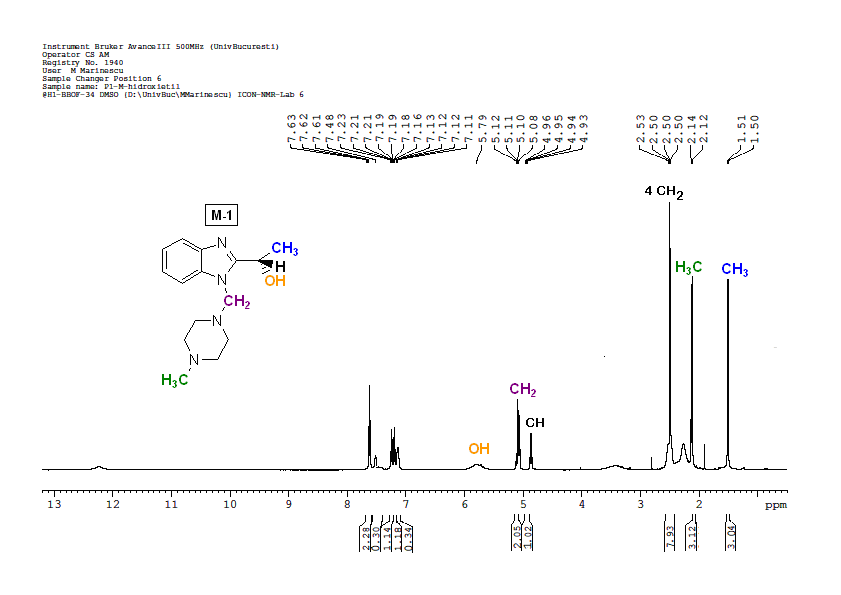


**Fig. S7.** ^1^H NMR spectra of Mannich base **M-1**.


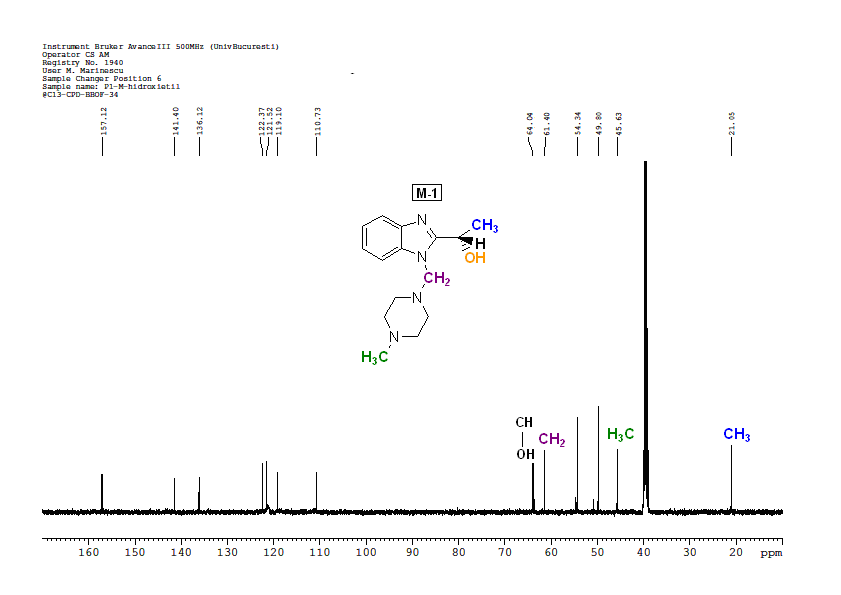


**Fig. S8.** ^13^C NMR spectra of Mannich base **M-1**.


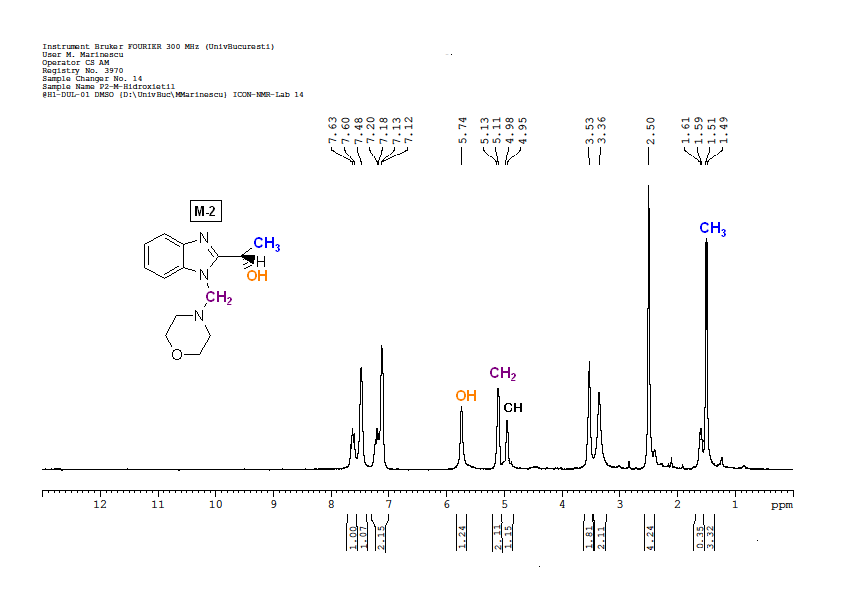


**Fig.S9.** ^1^H NMR spectra of Mannich base **M-2**.


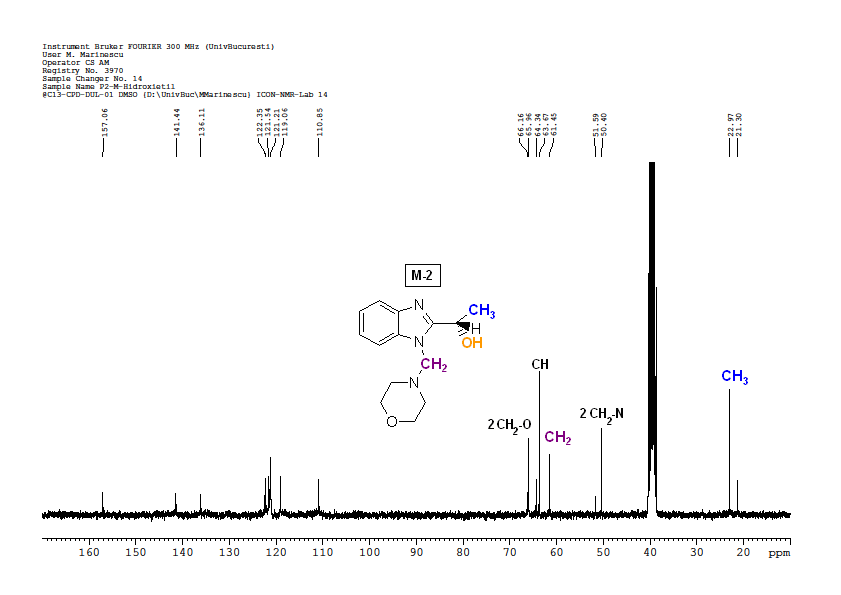


**Fig.S10.** ^13^C NMR spectra of Mannich base **M-2**.


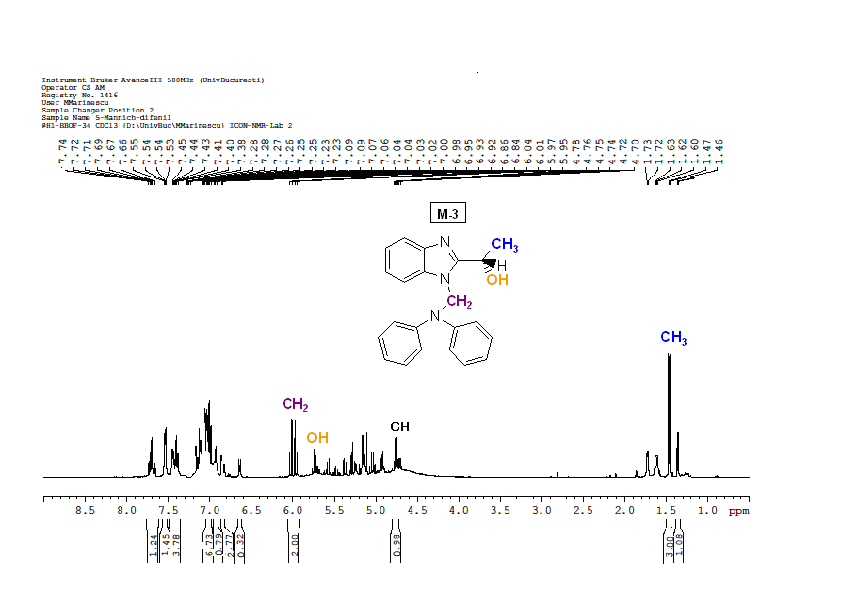


**Fig.S11.** ^1^H NMR spectra of Mannich base **M-3**.


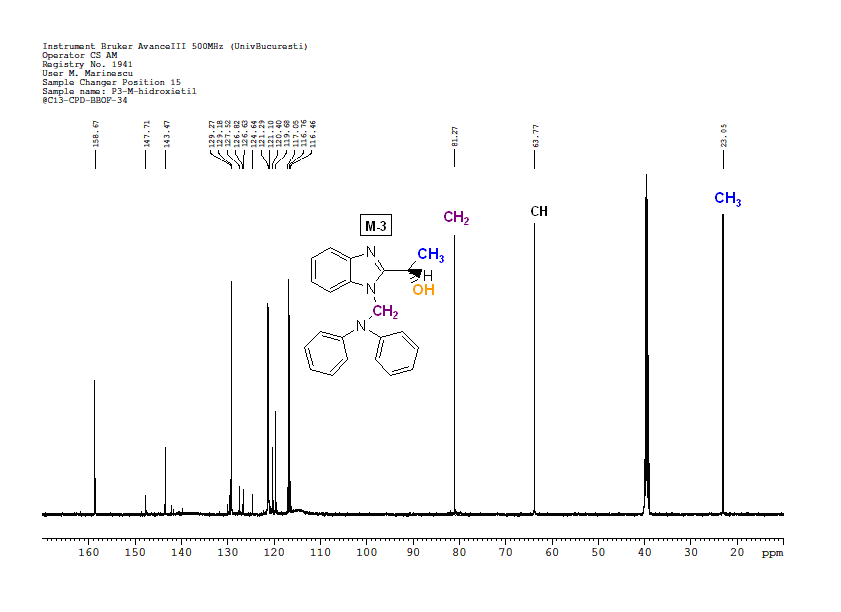


**Fig.S12.** ^13^C NMR spectra of Mannich base **M-3**.


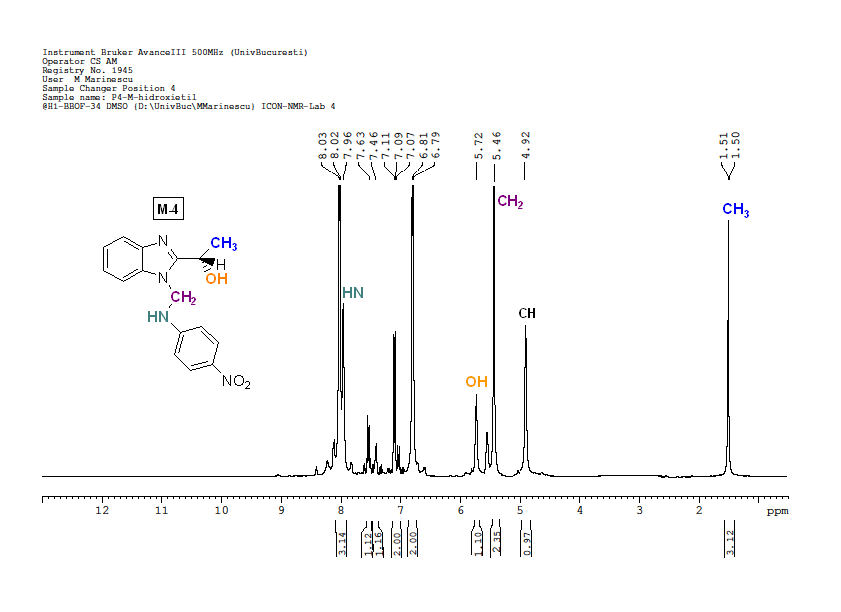


**Fig.S13.** ^1^H NMR spectra of Mannich base **M-4**.


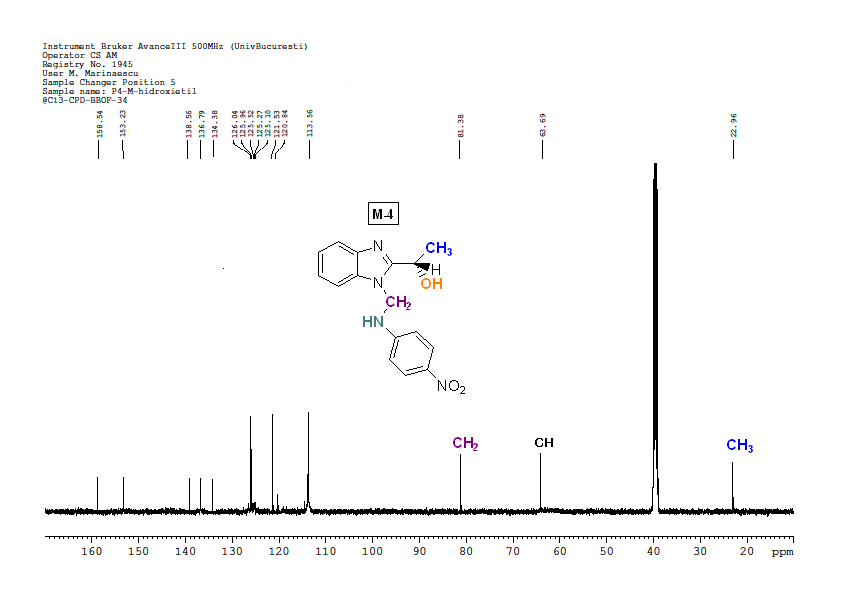


**Fig.S14.** ^13^C NMR spectra of Mannich base **M-4**.


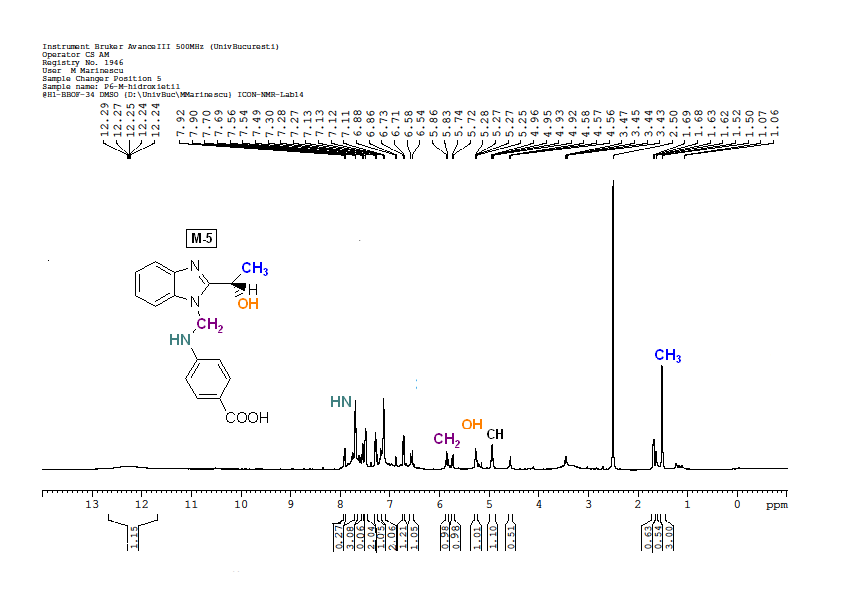


**Fig.S15.** ^1^H NMR spectra of Mannich base **M-5**.


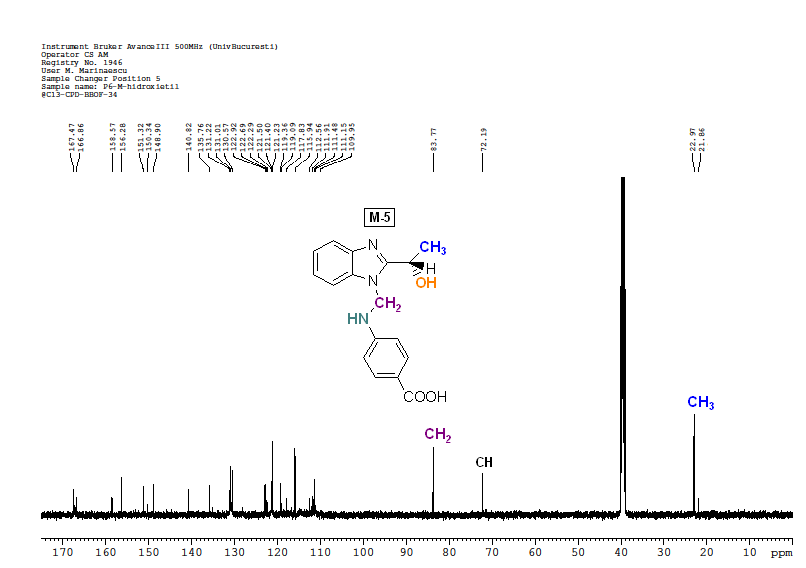


**Fig.S16.** ^13^C NMR spectra of Mannich base **M-5**.
